# Diaqua­(5-carb­oxy­benzene-1,3-dicarboxyl­ato-κ*O* ^1^)[8-ethyl-5-oxo-2-(piperazin-4-ium-1-yl)-5,8-dihydro­pyrido[2,3-*d*]pyrimidine-6-carboxyl­ato-κ^2^ *O* ^5^,*O* ^6^]zinc monohydrate

**DOI:** 10.1107/S1600536813002122

**Published:** 2013-01-31

**Authors:** Zhong-Li Ye, Guang-Hua Xin, Fu-Tian Zhang, Dong-Rong Xiao

**Affiliations:** a College of Chemistry and Chemical Engineering, Southwest University, Chongqing 400715, People’s Republic of China

## Abstract

In the title compound, [Zn(C_14_H_17_N_5_O_3_)(C_9_H_4_O_6_)(H_2_O)_2_]·H_2_O, the complex mol­ecule exists in a zwitterionic form. The Zn^II^ ion exhibits a distorted tetra­gonal-pyramidal geometry, being coordinated by two O atoms from the zwitterionic 8-ethyl-5-oxo-2-(piperazin-4-ium-1-yl)-5,8-dihydro­pyrido[2,3-*d*]pyrimidine-6-carboxyl­ate (*L*) ligand, one O atom from the 5-carb­oxy­benzene-1,3-dicarboxyl­ate dianion, [H*btc*]^2−^, and two O atoms from two aqua ligands. In the crystal, N—H⋯O and O—H⋯O hydrogen bonds link the components into a three-dimensional structure. The crystal packing exhibits π–π inter­actions between the aromatic rings, with centroid–centroid distances in the range 3.466 (3)–3.667 (3) Å.

## Related literature

For general background to the use of quinolones in the treatment of infections, see: Mizuki *et al.* (1996 ▶). For the crystal structure of a related compound, see: Zhang *et al.* (2011 ▶).
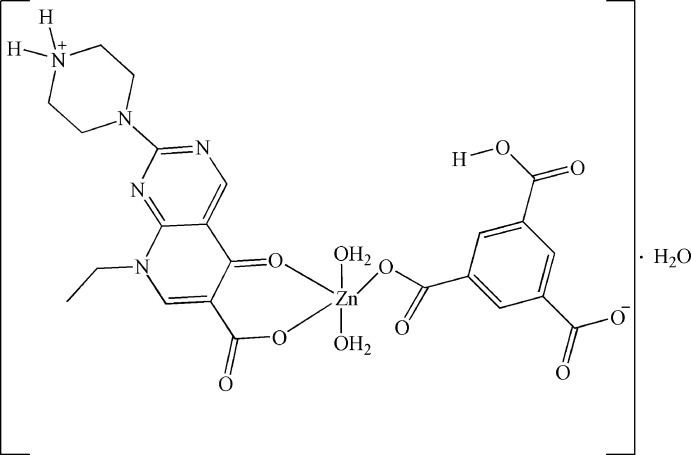


## Experimental

### 

#### Crystal data

[Zn(C_14_H_17_N_5_O_3_)(C_9_H_4_O_6_)(H_2_O)_2_]·H_2_O
*M* *_r_* = 630.87
Monoclinic, 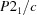

*a* = 13.5019 (11) Å
*b* = 12.5743 (10) Å
*c* = 17.7314 (10) Å
β = 125.575 (4)°
*V* = 2448.5 (3) Å^3^
*Z* = 4
Mo *K*α radiation
μ = 1.08 mm^−1^
*T* = 293 K
0.42 × 0.38 × 0.35 mm

#### Data collection

Bruker APEX CCD area-detector diffractometer
Absorption correction: multi-scan (*SADABS*; Bruker, 2001 ▶) *T*_min_ = 0.659, *T*_max_ = 0.703
12140 measured reflections
4299 independent reflections
3894 reflections with *I* > 2σ(*I*)
*R*_int_ = 0.019

#### Refinement

*R*[*F* ^2^ > 2σ(*F* ^2^)] = 0.028
*wR*(*F* ^2^) = 0.105
*S* = 0.86
4299 reflections
370 parameters
H-atom parameters constrained
Δρ_max_ = 0.30 e Å^−3^
Δρ_min_ = −0.29 e Å^−3^

### 

Data collection: *SMART* (Bruker, 2001 ▶); cell refinement: *SAINT* (Bruker, 2001 ▶); data reduction: *SAINT*; program(s) used to solve structure: *SHELXS97* (Sheldrick, 2008 ▶); program(s) used to refine structure: *SHELXL97* (Sheldrick, 2008 ▶); molecular graphics: *SHELXTL* (Sheldrick, 2008 ▶); software used to prepare material for publication: *SHELXL97*.

## Supplementary Material

Crystal structure: contains datablock(s) I, global. DOI: 10.1107/S1600536813002122/cv5383sup1.cif

Structure factors: contains datablock(s) I. DOI: 10.1107/S1600536813002122/cv5383Isup2.hkl

Additional supplementary materials:  crystallographic information; 3D view; checkCIF report

## Figures and Tables

**Table 1:** Hydrogen-bond geometry (Å, °)

*D*—H⋯*A*	*D*—H	H⋯*A*	*D*⋯*A*	*D*—H⋯*A*
N1—H1*A*⋯O2^i^	0.90	1.96	2.821 (2)	160
N1—H1*B*⋯O8^ii^	0.90	2.20	2.930 (2)	138
O9—H9*A*⋯O7^iii^	0.85	1.76	2.5722 (19)	158
O*W*1—H*W*1*A*⋯O5^ii^	0.82	1.83	2.647 (2)	176
O*W*2—H*W*2*A*⋯O6^ii^	0.82	1.85	2.674 (2)	174
O*W*3—H*W*3*A*⋯O3^iv^	0.84	2.27	2.977 (2)	142
O*W*3—H*W*3*B*⋯O*W*1^v^	0.84	2.37	3.159 (2)	156
O*W*2—H*W*2*B*⋯O2^vi^	0.83	1.89	2.715 (2)	169

Symmetry codes: (i) 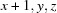; (ii) 
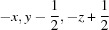
; (iii) 
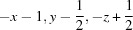
; (iv) 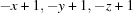; (v) 
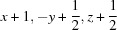
; (vi) 
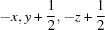
.

**Fig. 1.**
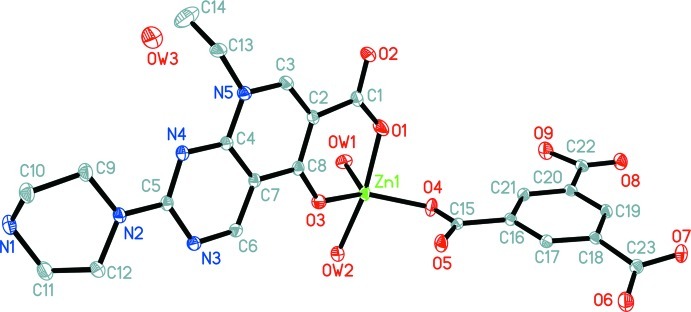
View of the title compound showing the atomic numbering and 30% probability displacement ellipsoids. H atoms have been omitted for clarity.

## supplementary crystallographic information

### Comment

Pipemidic acid (8-ethyl-5-oxo-2-piperazin-1-yl-5,8-dihydropyrido[2,3-*d*]pyrimidine-6- carboxylic acid), *L*, is a member of quinolones used to treat various infections (Mizuki *et al.*, 1996). The complexes of the *L* ligand and [H*btc*]^2-^ anion have not been reported till now. In this paper, we report the crystal structure of the title compound.

The asymmetric unit of the title compound is composed of one Zn^II^ ion, one *L* ligand , one [H*btc*]^2-^ anion (H_3_*btc* = benzene-1,3,5-tricarboxylic acid), two coordinated and one lattice water molecules (Fig. 1). All bond lengths in *L* are normal, though slightly different from those reported for base molecule *L* earlier by Zhang *et al.* (2011). So, the C1—O2, C3—O3 and C1—O1 bond lengths are 1.255 (2), 1.275 (2) and 1.255 (2) Å, respectively, versus 1.219 (2), 1.268 (3) and 1.319 (3) Å reported by Zhang *et al.* (2011).

In the crystal, intermolecular N—H···O and O—H···O hydrogen bonds (Table 1) link all moieties into three-dimensional supramolecular structure. The crystal packing exhibits π–π interactions between the aromatic rings with the intercentroids distances covering the range 3.466 (3) – 3.667 (3) Å.

### Experimental

A mixture of Zn(OAC)_2_^.^3H_2_O (0.546 g, 0.25 mmol), *L* (0.758 g, 0.25 mmol), H_3_*btc(*0.526 g, 0.25 mmol) and distilled water (8 mL) was stirred for 20 min in air. The mixture was then transferred to a 23 ml Teflon-lined hydrothermal bomb. The bomb was kept at 423 K for 96 h under autogenous pressure. Upon cooling, colorless block of 1 was obtained from the reaction mixture.

### Refinement

C-bound H atoms were positioned geometrically [C—H = 0.97 Å] and refined using a riding model approximation, with *U*_iso_(H) = 1.2 – 1.5 *U*_eq_(C). The N- and O-bound H atoms were located on a difference Fourier map, but placed in idealized positions [N—H = 0.90 Å, O—H = 0.82-0.85 Å] and refined as riding, with *U*_iso_(H) =1.2 U_eq_ of the parent atom.

### Crystal data

**Table:**

[Zn(C_14_H_17_N_5_O_3_)(C_9_H_4_O_6_)(H_2_O)_2_]·H_2_O	*F*(000) = 1304
*M**_r_* = 630.87	*D*_x_ = 1.711 Mg m^−^^3^
Monoclinic, *P*2_1_/*c*	Mo *K*α radiation, λ = 0.71073 Å
Hall symbol: -P 2ybc	Cell parameters from 12140 reflections
*a* = 13.5019 (11) Å	θ = 1.9–25.0°
*b* = 12.5743 (10) Å	µ = 1.08 mm^−^^1^
*c* = 17.7314 (10) Å	*T* = 293 K
β = 125.575 (4)°	Block, colourless
*V* = 2448.5 (3) Å^3^	0.42 × 0.38 × 0.35 mm
*Z* = 4	

### Data collection

**Table:**

Bruker APEX CCD area-detector diffractometer	4299 independent reflections
Radiation source: fine-focus sealed tube	3894 reflections with *I* > 2σ(*I*)
Graphite monochromator	*R*_int_ = 0.019
phi and ω scans	θ_max_ = 25.0°, θ_min_ = 1.9°
Absorption correction: multi-scan (*SADABS*; Bruker, 2001)	*h* = −16→13
*T*_min_ = 0.659, *T*_max_ = 0.703	*k* = −14→10
12140 measured reflections	*l* = −19→21

### Refinement

**Table:**

Refinement on *F*^2^	Primary atom site location: structure-invariant direct methods
Least-squares matrix: full	Secondary atom site location: difference Fourier map
*R*[*F*^2^ > 2σ(*F*^2^)] = 0.028	Hydrogen site location: inferred from neighbouring sites
*wR*(*F*^2^) = 0.105	H-atom parameters constrained
*S* = 0.86	*w* = 1/[σ^2^(*F*_o_^2^) + (0.105*P*)^2^] where *P* = (*F*_o_^2^ + 2*F*_c_^2^)/3
4299 reflections	(Δ/σ)_max_ = 0.001
370 parameters	Δρ_max_ = 0.30 e Å^−^^3^
0 restraints	Δρ_min_ = −0.29 e Å^−^^3^

### Special details

**Table:**

Geometry. All e.s.d.'s (except the e.s.d. in the dihedral angle between two l.s. planes) are estimated using the full covariance matrix. The cell e.s.d.'s are taken into account individually in the estimation of e.s.d.'s in distances, angles and torsion angles; correlations between e.s.d.'s in cell parameters are only used when they are defined by crystal symmetry. An approximate (isotropic) treatment of cell e.s.d.'s is used for estimating e.s.d.'s involving l.s. planes.
Refinement. Refinement of *F*^2^ against ALL reflections. The weighted *R*-factor *wR* and goodness of fit *S* are based on *F*^2^, conventional *R*-factors *R* are based on *F*, with *F* set to zero for negative *F*^2^. The threshold expression of *F*^2^ > σ(*F*^2^) is used only for calculating *R*-factors(gt) *etc*. and is not relevant to the choice of reflections for refinement. *R*-factors based on *F*^2^ are statistically about twice as large as those based on *F*, and *R*- factors based on ALL data will be even larger.

### Fractional atomic coordinates and isotropic or equivalent isotropic displacement parameters (Å^2^)

**Table:**

	*x*	*y*	*z*	*U*_iso_*/*U*_eq_	
Zn1	−0.032037 (19)	0.542603 (16)	0.212780 (15)	0.02453 (12)	
O1	−0.00857 (14)	0.45417 (12)	0.31957 (11)	0.0390 (4)	
OW1	−0.14163 (14)	0.42573 (12)	0.12210 (10)	0.0334 (3)	
HW1A	−0.1043	0.3693	0.1375	0.040*	
HW1B	−0.1824	0.4383	0.0659	0.040*	
N1	0.82562 (16)	0.33637 (14)	0.38179 (12)	0.0336 (4)	
H1A	0.8906	0.3303	0.3799	0.040*	
H1B	0.7758	0.2812	0.3501	0.040*	
C1	0.07412 (17)	0.39071 (15)	0.37520 (13)	0.0248 (4)	
OW2	−0.03717 (14)	0.61280 (11)	0.10620 (10)	0.0345 (3)	
HW2A	0.0169	0.5959	0.1002	0.041*	
HW2B	−0.0530	0.6774	0.0963	0.041*	
O2	0.06097 (13)	0.32612 (11)	0.42274 (10)	0.0340 (3)	
N2	0.68419 (14)	0.42975 (13)	0.42967 (11)	0.0244 (4)	
C2	0.19476 (16)	0.39307 (14)	0.38844 (12)	0.0219 (4)	
C2O	−0.37837 (16)	0.76072 (14)	0.26753 (12)	0.0220 (4)	
OW3	0.77949 (16)	0.23797 (14)	0.71317 (12)	0.0509 (4)	
HW3A	0.8273	0.2843	0.7177	0.061*	
HW3B	0.7854	0.1827	0.6896	0.061*	
O3	0.15171 (13)	0.53636 (10)	0.28385 (10)	0.0284 (3)	
N3	0.50374 (15)	0.52444 (12)	0.35526 (11)	0.0248 (4)	
C3	0.28179 (17)	0.32237 (15)	0.45016 (13)	0.0249 (4)	
H3A	0.2610	0.2759	0.4797	0.030*	
O4	−0.13831 (13)	0.64086 (11)	0.21967 (10)	0.0324 (3)	
N4	0.54190 (14)	0.37213 (12)	0.45188 (10)	0.0220 (3)	
C4	0.42950 (16)	0.38277 (14)	0.42995 (12)	0.0197 (4)	
O5	0.01241 (12)	0.74802 (11)	0.31946 (10)	0.0309 (3)	
N5	0.39455 (14)	0.31466 (12)	0.47158 (10)	0.0239 (3)	
C5	0.57379 (17)	0.44147 (14)	0.41227 (12)	0.0210 (4)	
O6	−0.12766 (16)	1.06302 (13)	0.42602 (13)	0.0456 (4)	
C6	0.39329 (18)	0.53030 (14)	0.33426 (13)	0.0226 (4)	
H6A	0.3426	0.5842	0.2943	0.027*	
O7	−0.28043 (13)	1.03074 (11)	0.43593 (10)	0.0290 (3)	
C7	0.34660 (17)	0.46027 (13)	0.36832 (12)	0.0196 (4)	
O8	−0.58074 (13)	0.74380 (12)	0.22537 (10)	0.0338 (3)	
C8	0.22443 (18)	0.46717 (14)	0.34316 (13)	0.0212 (4)	
O9	−0.51412 (13)	0.62674 (12)	0.16984 (10)	0.0361 (4)	
H9A	−0.5883	0.6067	0.1402	0.054*	
C9	0.76274 (19)	0.34109 (17)	0.48758 (14)	0.0303 (4)	
H9B	0.7156	0.2759	0.4681	0.036*	
H9C	0.7943	0.3543	0.5518	0.036*	
C10	0.86758 (18)	0.32865 (19)	0.47921 (14)	0.0346 (5)	
H10A	0.9277	0.3835	0.5154	0.042*	
H10B	0.9062	0.2602	0.5042	0.042*	
C11	0.7598 (2)	0.43770 (19)	0.33365 (17)	0.0377 (5)	
H11A	0.6851	0.4210	0.2739	0.045*	
H11B	0.8102	0.4807	0.3231	0.045*	
C12	0.73005 (19)	0.49977 (17)	0.39075 (16)	0.0329 (5)	
H12A	0.8026	0.5359	0.4406	0.040*	
H12B	0.6692	0.5532	0.3522	0.040*	
C13	0.48182 (19)	0.23716 (16)	0.54400 (14)	0.0321 (5)	
H13A	0.5642	0.2582	0.5676	0.039*	
H13B	0.4743	0.2393	0.5951	0.039*	
C14	0.4609 (3)	0.1272 (2)	0.5085 (2)	0.0662 (9)	
H14A	0.5191	0.0806	0.5575	0.099*	
H14B	0.4700	0.1242	0.4588	0.099*	
H14C	0.3801	0.1053	0.4862	0.099*	
C15	−0.09348 (16)	0.71577 (14)	0.27897 (12)	0.0215 (4)	
C16	−0.17983 (16)	0.76464 (14)	0.29685 (12)	0.0212 (4)	
C17	−0.15124 (17)	0.85606 (15)	0.35071 (12)	0.0212 (4)	
H17A	−0.0747	0.8870	0.3796	0.025*	
C18	−0.23701 (16)	0.90140 (14)	0.36141 (12)	0.0212 (4)	
C19	−0.34947 (17)	0.85178 (15)	0.32052 (12)	0.0234 (4)	
H19A	−0.4060	0.8802	0.3289	0.028*	
C21	−0.29326 (16)	0.71805 (15)	0.25523 (13)	0.0233 (4)	
H21A	−0.3129	0.6577	0.2187	0.028*	
C22	−0.50197 (17)	0.70998 (15)	0.21896 (12)	0.0244 (4)	
C23	−0.21103 (17)	1.00653 (16)	0.41192 (13)	0.0249 (4)	

### Atomic displacement parameters (Å^2^)

**Table:**

	*U*^11^	*U*^22^	*U*^33^	*U*^12^	*U*^13^	*U*^23^
Zn1	0.01950 (17)	0.02615 (18)	0.03055 (17)	0.00258 (8)	0.01604 (13)	−0.00079 (8)
O1	0.0278 (8)	0.0537 (10)	0.0451 (9)	0.0111 (6)	0.0268 (8)	0.0178 (7)
OW1	0.0390 (9)	0.0277 (7)	0.0291 (7)	−0.0004 (6)	0.0173 (7)	0.0011 (6)
N1	0.0305 (10)	0.0413 (10)	0.0381 (10)	0.0043 (8)	0.0251 (8)	−0.0010 (8)
C1	0.0241 (10)	0.0273 (10)	0.0284 (10)	−0.0039 (8)	0.0183 (9)	−0.0062 (8)
OW2	0.0375 (8)	0.0330 (8)	0.0440 (8)	0.0068 (6)	0.0300 (7)	0.0054 (6)
O2	0.0332 (8)	0.0350 (8)	0.0461 (9)	0.0001 (6)	0.0299 (7)	0.0057 (7)
N2	0.0200 (8)	0.0278 (8)	0.0292 (8)	0.0033 (7)	0.0165 (7)	0.0035 (7)
C2	0.0218 (9)	0.0231 (9)	0.0246 (9)	−0.0021 (7)	0.0156 (8)	−0.0042 (7)
C2O	0.0194 (9)	0.0243 (9)	0.0236 (9)	0.0008 (7)	0.0133 (8)	0.0039 (7)
OW3	0.0491 (11)	0.0523 (11)	0.0622 (11)	0.0057 (8)	0.0385 (9)	0.0089 (8)
O3	0.0205 (7)	0.0287 (8)	0.0363 (8)	0.0047 (5)	0.0166 (7)	0.0079 (6)
N3	0.0217 (9)	0.0237 (8)	0.0308 (9)	0.0016 (6)	0.0162 (7)	0.0034 (7)
C3	0.0257 (10)	0.0262 (10)	0.0272 (10)	−0.0032 (8)	0.0179 (8)	−0.0015 (8)
O4	0.0277 (8)	0.0298 (7)	0.0427 (8)	0.0010 (6)	0.0221 (7)	−0.0108 (6)
N4	0.0194 (8)	0.0240 (8)	0.0234 (8)	0.0011 (6)	0.0128 (7)	−0.0003 (6)
C4	0.0202 (9)	0.0204 (9)	0.0184 (8)	−0.0010 (7)	0.0111 (7)	−0.0027 (7)
O5	0.0215 (7)	0.0334 (8)	0.0406 (8)	−0.0005 (6)	0.0196 (7)	−0.0063 (6)
N5	0.0217 (8)	0.0249 (8)	0.0258 (8)	0.0011 (6)	0.0143 (7)	0.0030 (6)
C5	0.0198 (10)	0.0222 (9)	0.0217 (9)	−0.0009 (7)	0.0124 (8)	−0.0043 (7)
O6	0.0497 (11)	0.0391 (9)	0.0693 (12)	−0.0188 (8)	0.0467 (10)	−0.0230 (8)
C6	0.0214 (10)	0.0214 (9)	0.0252 (9)	0.0008 (7)	0.0137 (8)	0.0007 (7)
O7	0.0244 (8)	0.0345 (8)	0.0291 (7)	0.0061 (6)	0.0161 (7)	−0.0030 (6)
C7	0.0183 (10)	0.0201 (9)	0.0200 (9)	−0.0011 (6)	0.0109 (8)	−0.0034 (6)
O8	0.0228 (7)	0.0394 (8)	0.0424 (8)	0.0011 (6)	0.0208 (7)	−0.0019 (7)
C8	0.0201 (10)	0.0210 (9)	0.0232 (9)	−0.0016 (7)	0.0130 (8)	−0.0049 (7)
O9	0.0242 (8)	0.0423 (9)	0.0443 (9)	−0.0121 (6)	0.0213 (7)	−0.0137 (7)
C9	0.0251 (10)	0.0382 (12)	0.0309 (10)	0.0078 (8)	0.0182 (9)	0.0057 (9)
C10	0.0248 (11)	0.0461 (13)	0.0335 (11)	0.0086 (9)	0.0173 (9)	0.0055 (9)
C11	0.0343 (12)	0.0461 (13)	0.0431 (13)	0.0048 (10)	0.0284 (11)	0.0065 (10)
C12	0.0284 (11)	0.0321 (11)	0.0469 (12)	0.0013 (9)	0.0268 (10)	0.0054 (10)
C13	0.0305 (11)	0.0338 (11)	0.0332 (11)	0.0074 (9)	0.0192 (9)	0.0138 (9)
C14	0.096 (2)	0.0375 (14)	0.0573 (17)	0.0229 (15)	0.0402 (17)	0.0114 (13)
C15	0.0228 (10)	0.0189 (9)	0.0234 (9)	0.0048 (7)	0.0139 (8)	0.0058 (7)
C16	0.0182 (9)	0.0237 (9)	0.0228 (9)	0.0042 (7)	0.0125 (8)	0.0045 (7)
C17	0.0175 (9)	0.0233 (9)	0.0211 (9)	0.0025 (7)	0.0104 (7)	0.0034 (7)
C18	0.0219 (9)	0.0234 (9)	0.0201 (9)	0.0030 (7)	0.0132 (8)	0.0035 (7)
C19	0.0218 (9)	0.0283 (10)	0.0242 (9)	0.0049 (8)	0.0157 (8)	0.0039 (7)
C21	0.0230 (9)	0.0216 (9)	0.0263 (9)	−0.0005 (7)	0.0150 (8)	−0.0010 (7)
C22	0.0209 (9)	0.0284 (10)	0.0250 (9)	−0.0001 (8)	0.0139 (8)	0.0041 (8)
C23	0.0233 (10)	0.0261 (10)	0.0248 (9)	0.0042 (8)	0.0137 (8)	0.0028 (8)

### Geometric parameters (Å, º)

**Table:**

Zn1—O4	1.9505 (13)	O5—C15	1.237 (2)
Zn1—O3	2.0277 (14)	N5—C13	1.492 (2)
Zn1—OW1	2.0412 (14)	O6—C23	1.228 (3)
Zn1—OW2	2.0501 (14)	C6—C7	1.407 (3)
Zn1—O1	2.0553 (15)	C6—H6A	0.9300
O1—C1	1.255 (2)	O7—C23	1.271 (2)
OW1—HW1A	0.8193	C7—C8	1.439 (3)
OW1—HW1B	0.8271	O8—C22	1.210 (2)
N1—C10	1.475 (3)	O9—C22	1.310 (2)
N1—C11	1.503 (3)	O9—H9A	0.8547
N1—H1A	0.9000	C9—C10	1.515 (3)
N1—H1B	0.8999	C9—H9B	0.9700
C1—O2	1.255 (2)	C9—H9C	0.9700
C1—C2	1.505 (3)	C10—H10A	0.9700
OW2—HW2A	0.8247	C10—H10B	0.9700
OW2—HW2B	0.8319	C11—C12	1.506 (3)
N2—C5	1.343 (2)	C11—H11A	0.9700
N2—C12	1.460 (3)	C11—H11B	0.9700
N2—C9	1.468 (3)	C12—H12A	0.9700
C2—C3	1.368 (3)	C12—H12B	0.9700
C2—C8	1.431 (3)	C13—C14	1.477 (3)
C2O—C19	1.386 (3)	C13—H13A	0.9700
C2O—C21	1.396 (3)	C13—H13B	0.9700
C2O—C22	1.504 (3)	C14—H14A	0.9600
OW3—HW3A	0.8383	C14—H14B	0.9600
OW3—HW3B	0.8382	C14—H14C	0.9600
O3—C8	1.275 (2)	C15—C16	1.506 (2)
N3—C6	1.312 (3)	C16—C21	1.385 (3)
N3—C5	1.376 (2)	C16—C17	1.398 (3)
C3—N5	1.341 (2)	C17—C18	1.399 (3)
C3—H3A	0.9300	C17—H17A	0.9300
O4—C15	1.272 (2)	C18—C19	1.393 (3)
N4—C4	1.336 (2)	C18—C23	1.519 (3)
N4—C5	1.338 (2)	C19—H19A	0.9300
C4—N5	1.381 (2)	C21—H21A	0.9300
C4—C7	1.405 (3)		
			
O4—Zn1—O3	131.63 (6)	C22—O9—H9A	107.1
O4—Zn1—OW1	106.43 (6)	N2—C9—C10	110.23 (17)
O3—Zn1—OW1	121.73 (6)	N2—C9—H9B	109.6
O4—Zn1—OW2	98.83 (6)	C10—C9—H9B	109.6
O3—Zn1—OW2	87.86 (6)	N2—C9—H9C	109.6
OW1—Zn1—OW2	87.92 (6)	C10—C9—H9C	109.6
O4—Zn1—O1	90.56 (6)	H9B—C9—H9C	108.1
O3—Zn1—O1	85.95 (6)	N1—C10—C9	111.33 (16)
OW1—Zn1—O1	89.35 (6)	N1—C10—H10A	109.4
OW2—Zn1—O1	170.62 (6)	C9—C10—H10A	109.4
C1—O1—Zn1	131.15 (13)	N1—C10—H10B	109.4
Zn1—OW1—HW1A	109.7	C9—C10—H10B	109.4
Zn1—OW1—HW1B	118.8	H10A—C10—H10B	108.0
HW1A—OW1—HW1B	114.6	N1—C11—C12	111.01 (18)
C10—N1—C11	114.71 (17)	N1—C11—H11A	109.4
C10—N1—H1A	108.6	C12—C11—H11A	109.4
C11—N1—H1A	108.5	N1—C11—H11B	109.4
C10—N1—H1B	108.6	C12—C11—H11B	109.4
C11—N1—H1B	108.6	H11A—C11—H11B	108.0
H1A—N1—H1B	107.6	N2—C12—C11	110.94 (18)
O1—C1—O2	122.04 (18)	N2—C12—H12A	109.5
O1—C1—C2	119.83 (17)	C11—C12—H12A	109.5
O2—C1—C2	118.10 (17)	N2—C12—H12B	109.5
Zn1—OW2—HW2A	116.8	C11—C12—H12B	109.5
Zn1—OW2—HW2B	118.8	H12A—C12—H12B	108.0
HW2A—OW2—HW2B	111.3	C14—C13—N5	112.67 (19)
C5—N2—C12	122.99 (16)	C14—C13—H13A	109.1
C5—N2—C9	119.84 (16)	N5—C13—H13A	109.1
C12—N2—C9	117.14 (16)	C14—C13—H13B	109.1
C3—C2—C8	118.77 (17)	N5—C13—H13B	109.1
C3—C2—C1	117.39 (16)	H13A—C13—H13B	107.8
C8—C2—C1	123.80 (16)	C13—C14—H14A	109.5
C19—C2O—C21	119.41 (17)	C13—C14—H14B	109.5
C19—C2O—C22	121.32 (16)	H14A—C14—H14B	109.5
C21—C2O—C22	119.19 (17)	C13—C14—H14C	109.5
HW3A—OW3—HW3B	109.4	H14A—C14—H14C	109.5
C8—O3—Zn1	128.01 (13)	H14B—C14—H14C	109.5
C6—N3—C5	115.66 (16)	O5—C15—O4	123.77 (17)
N5—C3—C2	125.35 (17)	O5—C15—C16	121.28 (16)
N5—C3—H3A	117.3	O4—C15—C16	114.94 (16)
C2—C3—H3A	117.3	C21—C16—C17	119.49 (17)
C15—O4—Zn1	120.47 (12)	C21—C16—C15	117.82 (17)
C4—N4—C5	116.09 (16)	C17—C16—C15	122.66 (17)
N4—C4—N5	117.77 (16)	C16—C17—C18	120.43 (17)
N4—C4—C7	123.49 (17)	C16—C17—H17A	119.8
N5—C4—C7	118.74 (16)	C18—C17—H17A	119.8
C3—N5—C4	119.24 (15)	C19—C18—C17	118.99 (17)
C3—N5—C13	119.27 (16)	C19—C18—C23	120.31 (16)
C4—N5—C13	121.39 (16)	C17—C18—C23	120.61 (16)
N4—C5—N2	117.45 (17)	C2O—C19—C18	120.99 (17)
N4—C5—N3	125.89 (17)	C2O—C19—H19A	119.5
N2—C5—N3	116.65 (17)	C18—C19—H19A	119.5
N3—C6—C7	124.19 (17)	C16—C21—C2O	120.65 (17)
N3—C6—H6A	117.9	C16—C21—H21A	119.7
C7—C6—H6A	117.9	C2O—C21—H21A	119.7
C4—C7—C6	114.51 (17)	O8—C22—O9	124.50 (18)
C4—C7—C8	122.25 (17)	O8—C22—C2O	122.69 (18)
C6—C7—C8	123.24 (17)	O9—C22—C2O	112.82 (16)
O3—C8—C2	125.06 (18)	O6—C23—O7	124.09 (19)
O3—C8—C7	119.38 (17)	O6—C23—C18	119.57 (17)
C2—C8—C7	115.56 (16)	O7—C23—C18	116.33 (17)
			
O4—Zn1—O1—C1	−157.90 (19)	C3—C2—C8—O3	179.39 (18)
O3—Zn1—O1—C1	−26.19 (18)	C1—C2—C8—O3	−3.1 (3)
OW1—Zn1—O1—C1	95.68 (19)	C3—C2—C8—C7	−0.5 (2)
OW2—Zn1—O1—C1	22.6 (5)	C1—C2—C8—C7	176.99 (16)
Zn1—O1—C1—O2	−163.93 (14)	C4—C7—C8—O3	−177.04 (17)
Zn1—O1—C1—C2	17.9 (3)	C6—C7—C8—O3	3.5 (3)
O1—C1—C2—C3	179.80 (18)	C4—C7—C8—C2	2.8 (2)
O2—C1—C2—C3	1.6 (3)	C6—C7—C8—C2	−176.66 (16)
O1—C1—C2—C8	2.3 (3)	C5—N2—C9—C10	166.77 (17)
O2—C1—C2—C8	−175.91 (17)	C12—N2—C9—C10	−11.2 (3)
O4—Zn1—O3—C8	111.22 (16)	C11—N1—C10—C9	56.9 (2)
OW1—Zn1—O3—C8	−62.65 (17)	N2—C9—C10—N1	−44.5 (2)
OW2—Zn1—O3—C8	−148.87 (16)	C10—N1—C11—C12	−11.1 (3)
O1—Zn1—O3—C8	24.08 (16)	C5—N2—C12—C11	−120.9 (2)
C8—C2—C3—N5	−1.2 (3)	C9—N2—C12—C11	57.0 (2)
C1—C2—C3—N5	−178.84 (17)	N1—C11—C12—N2	−43.3 (2)
O3—Zn1—O4—C15	−2.41 (18)	C3—N5—C13—C14	79.6 (2)
OW1—Zn1—O4—C15	172.16 (14)	C4—N5—C13—C14	−104.1 (2)
OW2—Zn1—O4—C15	−97.42 (14)	Zn1—O4—C15—O5	17.8 (2)
O1—Zn1—O4—C15	82.66 (15)	Zn1—O4—C15—C16	−162.60 (12)
C5—N4—C4—N5	−178.14 (15)	O5—C15—C16—C21	−174.32 (17)
C5—N4—C4—C7	0.8 (3)	O4—C15—C16—C21	6.1 (2)
C2—C3—N5—C4	0.5 (3)	O5—C15—C16—C17	7.8 (3)
C2—C3—N5—C13	176.88 (18)	O4—C15—C16—C17	−171.76 (16)
N4—C4—N5—C3	−179.16 (16)	C21—C16—C17—C18	−1.0 (3)
C7—C4—N5—C3	1.8 (2)	C15—C16—C17—C18	176.78 (16)
N4—C4—N5—C13	4.6 (2)	C16—C17—C18—C19	2.4 (3)
C7—C4—N5—C13	−174.43 (16)	C16—C17—C18—C23	−173.99 (16)
C4—N4—C5—N2	−177.05 (16)	C21—C2O—C19—C18	0.2 (3)
C4—N4—C5—N3	3.2 (3)	C22—C2O—C19—C18	−176.69 (16)
C12—N2—C5—N4	179.90 (17)	C17—C18—C19—C2O	−2.0 (3)
C9—N2—C5—N4	2.1 (3)	C23—C18—C19—C2O	174.44 (16)
C12—N2—C5—N3	−0.3 (3)	C17—C16—C21—C2O	−0.8 (3)
C9—N2—C5—N3	−178.13 (16)	C15—C16—C21—C2O	−178.74 (16)
C6—N3—C5—N4	−4.5 (3)	C19—C2O—C21—C16	1.3 (3)
C6—N3—C5—N2	175.71 (17)	C22—C2O—C21—C16	178.18 (16)
C5—N3—C6—C7	1.9 (3)	C19—C2O—C22—O8	−2.0 (3)
N4—C4—C7—C6	−3.0 (3)	C21—C2O—C22—O8	−178.81 (18)
N5—C4—C7—C6	175.96 (16)	C19—C2O—C22—O9	177.94 (16)
N4—C4—C7—C8	177.49 (16)	C21—C2O—C22—O9	1.1 (2)
N5—C4—C7—C8	−3.6 (3)	C19—C18—C23—O6	−160.80 (19)
N3—C6—C7—C4	1.5 (3)	C17—C18—C23—O6	15.6 (3)
N3—C6—C7—C8	−178.97 (17)	C19—C18—C23—O7	18.2 (3)
Zn1—O3—C8—C2	−15.8 (3)	C17—C18—C23—O7	−165.44 (16)
Zn1—O3—C8—C7	164.05 (12)		

### Hydrogen-bond geometry (Å, º)

**Table:**

*D*—H···*A*	*D*—H	H···*A*	*D*···*A*	*D*—H···*A*
N1—H1*A*···O2^i^	0.90	1.96	2.821 (2)	160
N1—H1*B*···O8^ii^	0.90	2.20	2.930 (2)	138
O9—H9*A*···O7^iii^	0.85	1.76	2.5722 (19)	158
O*W*1—H*W*1*A*···O5^ii^	0.82	1.83	2.647 (2)	176
O*W*2—H*W*2*A*···O6^ii^	0.82	1.85	2.674 (2)	174
O*W*3—H*W*3*A*···O3^iv^	0.84	2.27	2.977 (2)	142
O*W*3—H*W*3*B*···O*W*1^v^	0.84	2.37	3.159 (2)	156
O*W*2—H*W*2*B*···O2^vi^	0.83	1.89	2.715 (2)	169

Symmetry codes: (i) *x*+1, *y*, *z*; (ii) −*x*, *y*−1/2, −*z*+1/2; (iii) −*x*−1, *y*−1/2, −*z*+1/2; (iv) −*x*+1, −*y*+1, −*z*+1; (v) *x*+1, −*y*+1/2, *z*+1/2; (vi) −*x*, *y*+1/2, −*z*+1/2.
